# Exploring the challenges of avoiding collisions with virtual pedestrians using a dual-task paradigm in individuals with chronic moderate to severe traumatic brain injury

**DOI:** 10.1186/s12984-024-01378-x

**Published:** 2024-05-16

**Authors:** Thiago de Aquino Costa Sousa, Isabelle J. Gagnon, Karen Z.H. Li, Bradford J. McFadyen, Anouk Lamontagne

**Affiliations:** 1 School of Physical & Occupational Therapy, McGill University, Montreal, QC Canada; 2 Feil and Oberfeld Research Centre, Jewish Rehabilitation Hospital – CISSS Laval, Site of the Centre for Interdisciplinary Research in Rehabilitation of Greater Montreal (CRIR), 3205 Place Alton-Goldbloom, Laval, QC H7V 1R2 Canada; 3 School of Rehabilitation Sciences, Université Laval, Quebec City, QC Canada; 4 Centre for Interdisciplinary Research in Rehabilitation and Social Integration (Cirris), CIUSSS Capitale Nationale, Quebec City, QC Canada; 5 Trauma/Child Development, Montreal Children’s Hospital, Montreal, QC Canada; 6 Department of Psychology, Concordia University, Montreal, QC Canada; 7 Centre for Research in Human Development, Concordia University, Montreal, QC Canada; 8 PERFORM Centre, Concordia University, Montreal, QC Canada

**Keywords:** Circumvention, Cognition, Gaze behaviour, Locomotion, Multitasking, Obstacle avoidance, Virtual reality

## Abstract

**Background:**

Individuals with a moderate-to-severe traumatic brain injury (m/sTBI), despite experiencing good locomotor recovery six months post-injury, face challenges in adapting their locomotion to the environment. They also present with altered cognitive functions, which may impact dual-task walking abilities. Whether they present collision avoidance strategies with moving pedestrians that are altered under dual-task conditions, however, remains unclear. This study aimed to compare between individuals with m/sTBI and age-matched control individuals: (1), the locomotor and cognitive costs associated with the concurrent performance of circumventing approaching virtual pedestrians (VRPs) while attending to an auditory-based cognitive task and; (2) gaze behaviour associated with the VRP circumvention task in single and dual-task conditions.

**Methodology:**

Twelve individuals with m/sTBI (age = 43.3 ± 9.5 yrs; >6 mo. post injury) and 12 healthy controls (CTLs) (age = 41.8 ± 8.3 yrs) were assessed while walking in a virtual subway station viewed in a head-mounted display. They performed a collision avoidance task with VRPs, as well as auditory-based cognitive tasks (pitch discrimination and auditory Stroop), both under single and dual-task conditions. Dual-task cost (DTC) for onset distance of trajectory deviation, minimum distance from the VRP, maximum lateral deviation, walking speed, gaze fixations and cognitive task accuracy were contrasted between groups using generalized estimating equations.

**Results:**

In contrast to CTLs who showed locomotor DTCs only, individuals with m/sTBI displayed both locomotor and cognitive DTCs. While both groups walked slower under dual-task conditions, only individuals with m/sTBI failed to modify their onset distance of trajectory deviation and maintained smaller minimum distances and smaller maximum lateral deviation compared to single-task walking. Both groups showed shorter gaze fixations on the approaching VRP under dual-task conditions, but this reduction was less pronounced in the individuals with m/sTBI. A reduction in cognitive task accuracy under dual-task conditions was found in the m/sTBI group only.

**Conclusion:**

Individuals with m/sTBI present altered locomotor and gaze behaviours, as well as altered cognitive performances, when executing a collision avoidance task involving moving pedestrians in dual-task conditions. Potential mechanisms explaining those alterations are discussed. Present findings highlight the compromised complex walking abilities in individuals with m/sTBI who otherwise present a good locomotor recovery.

## Background

Walking in the community is a daily living activity that is not a simple automated motor action. It requires a true “symbiosis” between sensorimotor, higher-level cognitive (e.g. executive function and attention) and cardiorespiratory systems [1, 2]. This complex activity also requires adaptations of the locomotor behavior in response to physical and social environmental factors [3]. Among the essential adaptations necessary for safe and independent community walking, obstacle avoidance is crucial, particularly in busy environments where other pedestrians must be avoided [4]. Successfully navigating such situations entails executing circumvention maneuvers. These collision avoidance maneuvers involve a temporary deviation to a new direction by adjustments over two phases: an anticipatory locomotor phase involving the initiation of lateral trajectory adjustments, and a clearance phase, where individuals ensure they have enough distance from the obstacle before proceeding to cross it [4–6]. 

Vision plays a crucial role in providing information about obstacle properties (nature, size, shape, etc.), location and motion characteristics (speed and direction), which are essential for successful obstacle circumvention [7–13]. Individuals continuously update information about the obstacle and compute the affordances of passable paths, adjusting their trajectory and speed to maintain a safe distance from the obstacle based on the real-time perception of a theoretical point of collision (TPC) [14, 15]. For instance, healthy individuals are expected to initiate the maneuver at least 1 m away from the obstacle [4], deviate laterally at a distance necessary to ensure clearance [5] and adjust their walking speed to adjust to spatial and temporal properties of the obstacle [16]. The literature also shows that circumvention strategies can be influenced by personal factors, such as older age and neurological conditions [17–21], as well as situational factors such as obstacle characteristics (nature, size, shape, etc.) and obstacle location, speed and direction of motion [17, 20, 22–25]. 

Obstacle circumvention becomes notably challenging in situations where attention is divided, such as in dual-task walking (e.g. walking and performing a cognitive task at the same time), increasing the risk of collisions [21] and falls [26]. If a motor task or a cognitive task demands more attentional resources or compete for the same attentional structures, exceeding an individual’s total attentional capacity [27], it can result in interference within one or both tasks, potentially leading to a decline in the performance of one or both tasks [28]. The few studies that have investigated the impact of dual tasking on the circumvention of moving obstacles (pedestrians or objects) in healthy young adults have found a cognitive interference, that is a deterioration in the cognitive task but no differences in the locomotor task in the dual- vs. single-task conditions [29–31]. In similar dual-task conditions, however, individuals with a neurological condition such as stroke typically present with mutual (cognitive-motor) interferences [21, 32]. 

Traumatic brain injury (TBI), a brain damage caused by an external mechanical force [33], is considered a leading cause of death and disability worldwide [34]. It can lead to sensorimotor and cognitive impairments [33] that negatively impact the completion of activities of daily living. The severity of a TBI can be classified as mild, moderate or severe, depending on both the presence and extent of signs such as loss or reduced consciousness, loss of memory, motor response, verbal response, eyes opening, disrupted vision, and abnormal findings on structural brain imaging [33]. Once rehabilitation is completed, individuals with moderate-to-severe TBI (m/sTBI) often present a good recovery of independent walking [35] but walking in the community remains compromised [36]. When it comes to complex walking tasks, such as stepping over an obstacle [37], hopping, or walking on irregular terrains [36, 38], limitations become even more obvious. Longitudinal studies that followed individuals with m/sTBI over time revealed a progressive improvement in functional independence within the first 12 months post-injury, but a subsequent decline between the 2nd and 7th years post-injury [39, 40]. 

Cognitive function in individuals with m/sTBI also generally improves in the first year post-injury, but a non-negligible proportion of individuals (27%) later experience a decline between 12 and 30 months post-injury [41]. Individuals with m/sTBI also struggle to divide their attention effectively between walking and a concurrent cognitive task [37, 38, 42], which also seems to be dependent on locomotor and cognitive task complexity. Vallée et al. (2006)^29^ observed that individuals with m/sTBI, when stepping over obstacles under dual-task conditions with varying levels of complexity, demonstrated mutual cognitive-locomotor dual-task interference for the more complex condition which involved the wider obstacle and a simultaneous Stroop word task.

While previous studies have allowed uncovering the presence of locomotor and cognitive deficits in individuals with m/sTBI, there is a paucity of research on complex locomotor tasks involving obstacle circumvention in this population, both under single and dual-task conditions. To date, only one unpublished study has investigated the strategies used by individuals with m/sTBI to circumvent obstacles while walking [43]. The authors compared a small high-functioning sample of 8 participants with m/sTBI (average time since the injury was 5 months) to a group of age-matched healthy individuals, as they circumvented a static or orthogonally-approaching cylinder with or without performing a visually-based Stroop-word task. Under dual-task conditions, individuals with m/sTBI presented slower response times and made more errors on the cognitive task compared to healthy individuals. They also showed slower walking speeds and larger obstacle clearances. The use of a visually-based cognitive task, however, might have impacted the performance on the obstacle circumvention task, as both tasks heavily rely on vision, thereby creating higher interference. 

Research also shows that attentional processes and gaze behaviour (where a person is looking) are closely linked at the neural level, making gaze allocation indicative of an individual’s focus of attention [44]. In recent locomotor studies, gaze fixation duration was shown to be modulated according to factors such as the location and/or direction of displacement of pedestrians present in the environment [8, 45], suggesting that visually acquiring information about the spatial properties of the obstacle plays a role in successful collision avoidance. Additionally, a study involving stepping over an obstacle while performing a cognitive task showed that individuals with m/sTBI, unlike healthy individuals, presented a larger cognitive dual-task cost when performing a visual vs. auditory-based cognitive task [42]. Although gaze behaviour was not measured in the mentioned study [42], the findings were interpreted as individuals with m/sTBI allocating greater visual attention to the obstacle during the avoidance task. A meta-analysis further revealed that individuals with TBI experience deficits in higher-order visual-spatial attentional processing, particularly in cases of moderate-to-severe and severe injury [46]. Characterizing gaze behaviour during obstacle circumvention and how it may be affected by the addition of a cognitive task may thus provide further insight into underlying mechanisms explaining dual-task walking abilities in individuals with m/sTBI.

To our knowledge, there is no research examining circumvention strategies of individuals with chronic m/sTBI avoiding pedestrians as interferers (like encountered when walking in the community), particularly using gaze behaviour as a marker of visual attention. Therefore, the first objective of this study was to compare individuals with m/sTBI to age-matched control individuals in relation to locomotor and cognitive costs during concurrent tasks of circumventing a virtual pedestrian (VRP) and responding to an auditory-based stimulus. The second objective was to characterize gaze behaviour associated with the circumvention task performed in single and dual-task conditions in both groups. In relation to the first objective, it was hypothesized that individuals with m/sTBI would present dual-task costs (DTCs) in both the locomotor and cognitive tasks, while the healthy individuals, based on previous work involving similar obstacle circumvention tasks [31, 47], would only show a small cognitive DTC. For the second objective, longer gaze fixations on the approaching VRPs were expected in the dual- vs. single task condition for both groups, but such difference would be more pronounced in the m/sTBI group.

## Methods

### Participants

This experimental study used a within-between repeated measure design. A convenience sample of 24 participants, divided equally into two groups involving healthy control participants and individuals with chronic m/sTBI, was recruited. The sample size was estimated (G*Power version 3.1.9.6) based on changes in ‘minimum distance’ from the obstacle previously documented as the main measure in a study using a similar paradigm in participants with stroke and healthy control participants [21]. Considering a significance level of 0.05 and a power of 80%, a sample size of 12 participants per group was recommended.

The m/sTBI participants were recruited from the discharge list of the Trauma rehabilitation program of the Jewish Rehabilitation Hospital (JRH) and via a local association supporting individuals with TBI and their family. Healthy controls were recruited from a convenience sample at the JRH, McGill University and from the general community.

To be eligible for participation in the study, individuals in the m/sTBI group had to meet the following inclusion criteria: have experienced a chronic TBI at least 6 months earlier; have had their brain injury classified as moderate or severe, based on meeting at least two of three of the following criteria: Glasgow Coma Scale (GCS) ≤ 12, post-traumatic amnesia (PTA) > 1 day, and abnormal brain image; [48] be aged between 18 and 55 years; be able to perform the 10 m Walk Test (10MWT) [49] at a speed of 0.7 m/s or greater [37, 50] without a walking aid and; have sufficient cognitive function to follow instructions and provide autonomous consent. The age cut-off of 55 years was selected in order to minimize the impact of older age and associated comorbidities on mobility and cognitive functions. For the healthy group, age and sex-matched participants with no known history of TBI or concussion and intact cognitive function (Montreal Cognitive Assessment (MoCA) score > 25) [51] were recruited. Individuals in both groups further had to receive primary education in either the English or French language, in order to avoid language barriers when performing the cognitive task. In addition, they had to present normal or corrected-to-normal visual (logMAR of 0.4 or greater on the ETDRS chart [52]), and auditory acuity (ability to correctly repeat an audio message played at 50dB through the head-mounted display (HMD) headset over 5 trials [23]). Exclusion criteria for both groups included any conditions that could interfere with locomotion, other than the TBI for the m/sTBI group.

Prior to the study, all participants provided written informed consent. The study was approved by the Research Ethics Board en réadaptation et en déficience physique of the CIUSSS du Centre-Sud-de-l’Île-de-Montréal.

### Experimental setup and procedures

The study comprised two evaluation sessions taking place on the same day, each lasting 2–2.5 h. In the first session, eligible participants underwent a clinical assessment of balance, mobility and cognition. In the second session, participants completed a comprehensive laboratory evaluation. The laboratory tasks consisted of: (1) a single walking task (ST walking); (2) a single cognitive task (cognitive ST) with two complexity levels (a simple pitch discrimination task and a complex Auditory Stroop task) and; (3) dual task conditions that combined the walking task with both complexity levels of the cognitive task, resulting in a simple (DT Simple) and a complex dual-task (DT Complex) condition. To minimize potential order and learning effects, the order of the tasks was randomized. Participants were offered breaks as needed, with a mandatory long break of approximately 1 h between evaluation sessions.

### Clinical assessment

First, participants were interviewed to obtain demographic data and information on the following: time since the onset of TBI and cause, determined using medical chart information and reconfirmed by administering The Ohio State University Traumatic Brain Injury Identification Method [53], level of education (i.e., number of years of school completed) and prior experience with immersive virtual reality (VR) environments (i.e., yes or no). Handedness was determined using the Edinburgh Handedness Inventory [54]. Overground walking speed was measured using the 10MWT [49] and ambulatory skills were characterized using the Community Balance and Mobility Scale (CB&M) [55]. The ability to dual task while walking and balance confidence were assessed using the Timed Up and Go Cognitive (TUG-Cog) [56] and Activities-specific Balance Confidence (ABC) Scale [57], respectively. Cognitive function was characterized using the Montreal Cognitive Assessment (MoCA), the Digit Span Test [58] as well as the Trail Making Test A (TMT-A) and B (TMT-B) [59]. 

### Single obstacle avoidance task

**Fig. 1:**
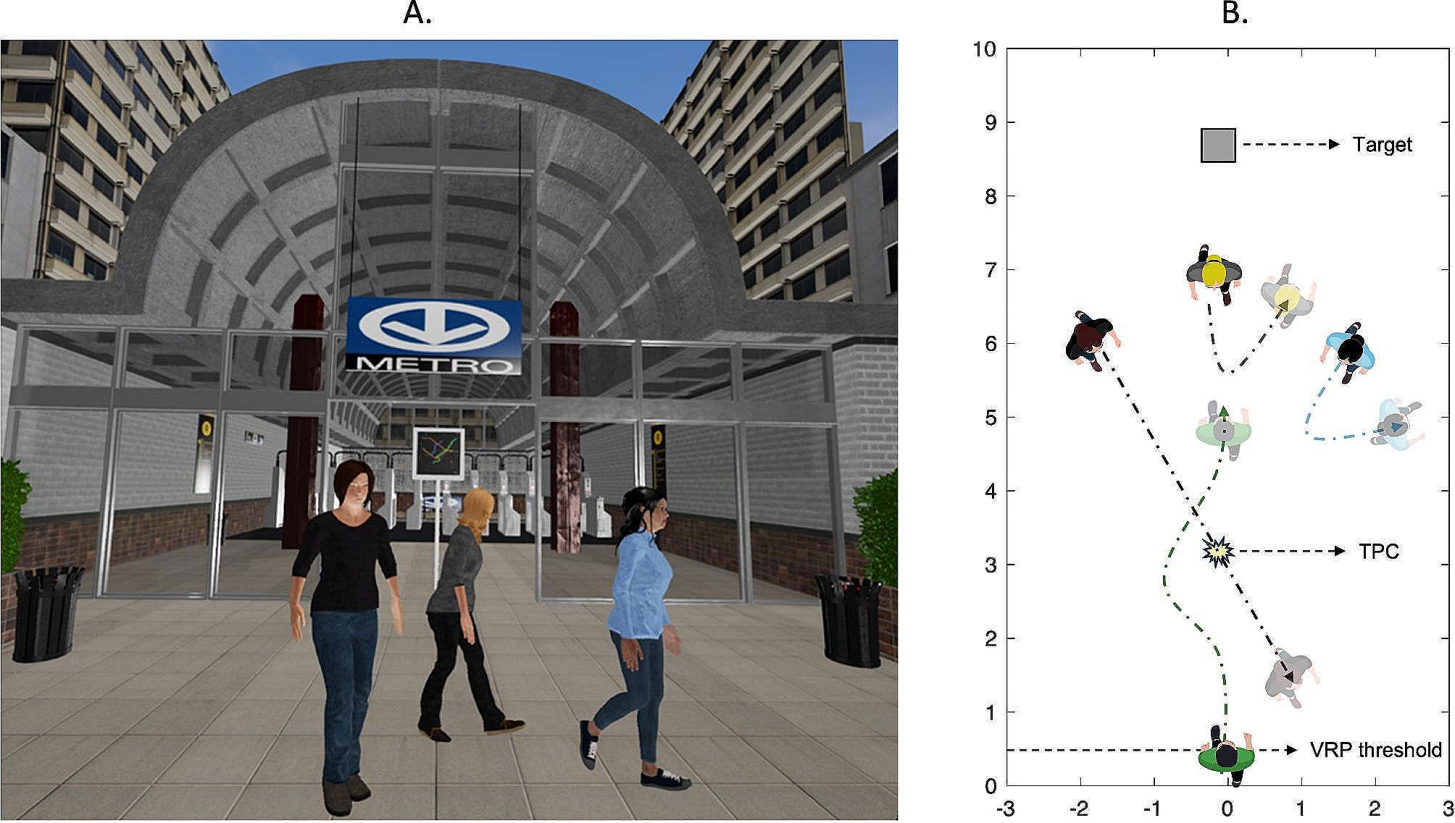
(**A**) Virtual environment representing a subway station with pedestrians, as viewed by the participants during a diagonal virtual pedestrian (VRP) approach (left). (**B**) Schematic representation of the obstacle circumvention task from a bird’s eye view, when avoiding a left VRP approach

Participants were assessed while walking overground and immersed in an ecological virtual environment representing a subway station in Montreal, Canada (Fig. 1-A), created in Autodesk Maya and controlled using the Unreal game engine 4.27.2. Participants were positioned at a designated starting position marked at one end of the walking area. They faced the target (Montreal subway map) located straight ahead (0°) in the far space (8.5 m). Three female (based on an age range of 35–45 years) VRPs acting as interferers were created using motion capture data from healthy female individuals of the same age range [6]. The VRPs were positioned in an arc fashion at 0° (straight ahead), 30° to the right and 30° to the left from a theoretical point of collision located 3.25 m in front of the participant, with a radius of 3 m. The theoretical point of collision is a point where a collision with an approaching VRP would occur if the participant does not perform any locomotor adjustments (Fig. 1-B).

The VRPs were non-reactive and walked with a neutral gait pattern at 1.2 m/s, replicating the average comfortable walking speed of healthy female adults [60]. The choice of female VRPs walking with a neutral gait pattern was guided by research indicating that gender and emotions of gait are factors that can influence perception on the part of the observers [61, 62]. The participants viewed the virtual environment using the HTC VIVE Pro Eye, an HMD (refresh rate of 90 Hz) that has an integrated binocular eye tracker and audio headset. The HMD is equipped with tracking sensors that provide information on the position and orientation of the head. This information was supplied in real-time to the Unreal game engine to update the camera view of the participants within the virtual scene according to their head position and orientation. The eye tracker within the HMD has an accuracy of 0.5 to 1.1° across the entire 110-degree field of view. Participants’ head, eye and gaze position data were recorded in Unreal at 90 Hz.

An initial calibration process was conducted to align the virtual and the physical environments, as per a procedure described earlier [6]. Calibration of the VIVE Pro Eye eye tracker was performed initially and repeated after every block of ten (10) walking trials, or at any point that the HMD was repositioned or removed from the participant’s head. During data collection, participants were instructed to begin walking at a comfortable speed towards the target after the words ‘Get ready’ disappeared from the screen, and to stop walking when seeing the word ‘Stop’. They were further instructed to avoid any collision with an approaching VRP if present. Once participants reached 0.5 m of forward walking, the three VRPs located in the far space started walking toward the theoretical point of collision. After taking one step, two of the VRPs turned around and walked away while the remaining one continued walking towards the theoretical point of collision.

Five VRP conditions were presented in a random order, including (1) a right and (2) left approach (± 30°), (3) a middle approach (0°), (4) an all-back condition where all VRPs turned around and walked away (catch trials) and (5) a control trial without any VRPs. Trials without VRPs served the purpose of evaluating the participants comfortable walking speed in the virtual environment and were used as a reference of straight-ahead trajectory to calculate the onset time/distance of trajectory deviation. In the event of a collision, the word ‘Collision’ flashed on the screen and the participants had to stop and walk back towards the starting point. Six trials for each of the 5 VRP directions were randomly performed, for a total of 30 trials.

### Single cognitive task

Participants were assessed while seated and observing the static virtual environment in the HMD. The cognitive task was an auditory pitch-discrimination task with 2 levels of complexity, for which sound stimuli were delivered through the audio headset of the HMD. In the simple task, the word “Cat” (or “Chat” in French) was presented in a high or low pitch while in the complex task, the words “High” or “Low” (“Haut” and “Bas” respectively in French) were presented in a high or low pitch (i.e., an Auditory Stroop Task). The Auditory Stroop or “High-Low” task is considered to be a more intricate task than the simple pitch discrimination task, especially when the trial presents an incongruent condition (e.g., word ‘high’ in low pitch), as greater attention and inhibition is required to correctly identify the pitch without being influenced by the meaning of the word [63]. The intensity of the sound stimulus was set to 70 dB, a level that was considered enough and comfortable based on a study associating TBI severity with audiometric measures [64]. 

Participants were instructed to verbally report the pitch of the words as accurately as possible (high or low) while ignoring the meaning of the words. The single cognitive task conditions, delivered in a random order, were tested by means of 6 trials (3 for the simple and 3 for the complex task) each lasting 50s. For each trial, multiple sound stimuli were delivered at variable interstimulus intervals lasting between 1.5 s and 1.9 s. The trial duration was based on the duration and number of the walking trials, considering an average adult walking speed of 1 m/s to 1.2 m/s [60], in order to get a similar number of auditory stimuli in the single vs. dual-task condition. The participants’ answers (including missed responses) were digitally recorded as well as noted by the experimenter for offline analysis.

### Dual-task conditions

The dual-task conditions required participants to perform simultaneously the walking (avoiding collision with a VRP) and the cognitive tasks (either simple or complex), resulting in the simple and complex DT conditions. The auditory cognitive task was presented at the same time intervals as in the single cognitive task conditions. Instructions given to participants were to walk towards the target and to avoid VRPs as needed while reporting the pitch of the words simultaneously. The DT conditions comprised 30 trials and their order of presentation was randomized.

The perception of difficulty for the single cognitive and dual-task conditions was assessed after each long single-task trials and after each block of 10 dual-task trials, with a numerical rating scale that ranged from 0 (no difficulty) to 10 (extreme difficulty). At the very end of the experiment, VR-related motion sickness was measured through the Fast Motion Sickness Scale [65] while the feeling of presence in the virtual environment was measured through the Single-Item Measure of Presence in VR [66]. 

### Data analysis

Data recorded in the Unreal engine were exported to Matlab (MathWorks, USA) and used to calculate the measures related to the locomotor and cognitive tasks. Because participants exhibited very similar behaviour when navigating VRP interferers approaching from the left vs. right, as also seen in other studies [45, 67, 68], data from the left and right VRP directions were combined into a single ‘diagonal’ condition. There was only one missing trial for one participant that was not recorded due to technical issues.

Locomotor measures for this study encompassed the number of collisions, minimum distance, walking speed (minimum, average, and maximum), onset distance, onset time and maximum lateral displacement. The number of collisions was calculated by counting the number of times the distance between the lateral edges of the participant and the VRP was less than the sum of the radii of the participant and VRP (42.5 cm). Minimum distance was calculated as the minimum distance maintained between the center of the participant’s head and the VRP’s centre of the neck over a time window spanning from when VRPs were triggered to walk to the point of VRP crossing, that is the point when the antero-posterior position of the participant was the same as that of the VRP. Walking speed was calculated using the first derivative of the participant’s head trajectory over a distance starting at 1 m of anteroposterior displacement of the participant (to avoid initial acceleration) until the point of VRP crossing. Minimum, mean and maximum walking speed values were then extracted. The onset distance of trajectory deviation was calculated as further detailed in Buhler et al. [67] Briefly, we first calculated the peak mediolateral displacement (ML) before the point of interferer crossing. Then, within a time window that spanned from the onset of VRP motion to the first point where participant’s ML displacement reached 25% of its peak value, a linear regression model was fitted to the data and then extrapolated until the point of VRP crossing. Finally, if the signal crossed the 99% confidence of this linear prediction, the onset of deviation and reorientation was obtained as the first preceding point at which the first derivative of the signal had a value smaller than zero (for a detailed schematic representation, see [67]). At this point, the onset distance of the trajectory deviation was calculated as the Euclidean distance between the participant and the VRP. As for onset time of trajectory deviation, it was calculated as the time between the onset distance of trajectory deviation and the point of VRP crossing. Maximum lateral displacement was defined as the maximum lateral excursion occurring in a window that spanned from the onset distance of trajectory deviation to the point of VRP crossing.

Gaze-related measures included the percent duration of gaze fixations directed toward objects of interest which included the approaching VRP, other VRPs, the goal, and the rest of the environment. Gaze fixation instances were identified for every data frame at which a participant’s gaze vector collided on the respective object of interest in a time window that began at the onset of VRP movement and ended at the point of VRP crossing. The total of instances of gaze fixation on a given object of interest was then expressed as a percentage of the total number of data frames in that time window. As it was initially far and often occluded by a VRP, the goal was considered as the combination of the subway map and subway entrance.

Response accuracy during the cognitive tasks was assessed by means of percent correct responses. These were calculated as the percentage of correct responses with respect to the total number of auditory stimuli. All measures that presented a significant main effect of task or a group by task interaction effect had their DTC calculated by the use of the following formula: DTC = 100 * (single-task score – dual-task score) / single-task score [69]. 

### Statistical analysis

Clinical assessment measures, perception of difficulty, presence and motion sickness questionnaires were compared between groups using two-sided independent-sample T-tests for continuous variables and two-sided Pearson Chi-square tests for categorical variables. Locomotor, gaze behaviour and cognitive measures were contrasted between groups and across tasks using generalized estimating equations (GEE). For the locomotor and gaze behaviour measures, our model incorporated an exchangeable correlation matrix, with two within-subject factors, namely direction of VRP approach (diagonal and middle) and task (ST walking, DT simple, and DT complex), along with one between-subject factor, which was the group (healthy and m/sTBI). As for the cognitive measure, the model featured one within-subject factor, that is the task (cognitive ST simple, cognitive ST complex, DT simple, and DT complex) and group as the between-subject factor. The DTCs were analyzed by means of a GEE model consisting of complexity (simple vs. complex) as the within-subject factor and group as the between-subject factor. Post-hoc comparisons were carried out when appropriate using least significant difference (LSD) with Bonferroni adjustments. When considering both groups, a total of 1295 walking trials with a VRP as an interferer were collected. Except for the number of collisions (*n* = 16), all measures were calculated using collision-free trials. Eight trials (four in the healthy group and four in the m/sTBI group) where participants circumvented the approaching VRP by passing in front of it (0.6% of trials) instead of behind (99.4% of trials) were also excluded from the analysis altogether. Additionally, trials without a trajectory deviation (*n* = 104, representing 16.4% of the total trials for the m/sTBI group and *n* = 17, representing 2.7% of the total trials for the control group) could not be represented in the analysis of onset distance and onset time of trajectory deviation, as well as maximum lateral deviation. In five trials (performed by 2 participants in the m/sTBI group), the point of VRP crossing happened within the acceleration area. Those trials were thus excluded from the analysis of walking speed (minimum, average, and maximum). Finally, gaze data from one healthy participant, identified as a statistical outlier, were also excluded from the analysis of gaze behaviour only. Statistical analyses were performed in SPSS Statistics 29.0.0.0 (241) with an alpha level of significance set to *p* < 0.05.

## Results

### Characteristics of participants

Table 1 provides an overview of the characteristics of participants from each group. No significant differences emerged between the groups concerning age, sex, handedness, level of education, prior experience with immersive VR environments, single and dual-task performance on the Timed Up and Go, memory assessed through the Digit Span test (both forward and backward), and self-reported sense of presence within the virtual environment, as measured by the Single-Item Measure of Presence. Individuals with m/sTBI, however, demonstrated alterations in their balance and mobility, as indicated by significantly reduced scores on the ABC and CB&M tests, and slower overground walking speed on the 10MWT compared to the healthy group. In terms of cognitive function, participants with m/sTBI scored significantly lower on the MoCA and showed longer completion times for TMT-A and TMT-B tests. Furthermore, they reported small but significantly higher motion sickness ratings (3.2 out of 20) on the Fast Motion Sickness Scale compared to the healthy group (0.2 out of 20).

**Table 1:** Characteristics of participants

	Group		*P*-value
	Healthy (*n* = 12)	m/sTBI (*n* = 12)	
**Demographics**			
Age (years)	41.8 (8.3)	43.3 (9.5)	0.685
Sex (Female/Male)†	4/8	3/9	0.653
Handedness (Left/Ambidextrous/Right) †	0/0/12	0/2/10	0.14
Level of education (years of schooling)	17.0 (2.6)	16.3 (5.6)	0.710
VR experience (Yes/No) †	6/6	8/4	0.408
TBI severity (Moderate/Severe) †	-	4/8	-
Time since TBI (months) ^§^	-	29 (49.8)	-
PTA duration (days) ^§^	-	10.5 (25.5)	-
GCS (3–15)	-	7.1 (3.6)	-
**Balance & Mobility**			
ABC (%)	98.5 (3.0)	76.2 (15.0)	**< 0.001**
CB&M (0–96)	88.4 (6.6)	63.4 (23.8)	**0.002**
Comfortable walking speed (m/s)	1.5 (0.2)	1.2 (0.2)	**0.032**
Maximum walking speed (m/s)	2.2 (0.3)	1.7 (0.4)	**< 0.001**
TUG (s)	8.3 (1.0)	9.3 (2.3)	0.168
TUG-Cog (s)	9.9 (2.0)	11.4 (3.3)	0.2
TUG-DTC (%)	-18.7 (18.0)	-21.4 (13.5)	0.692
**Cognition**			
MoCA (max = 30)	28.3 (1.5)	24.8 (3.2)	**0.002**
TMT-A (s)	22.7 (5.4)	40.1 (14.3)	**< 0.001**
TMT-B (s)	62.2 (24.7)	99.4 (40.1)	**0.012**
Digit Span - Forward (s)	10.0 (1.9)	9.1 (3.6)	0.444
- Backward (s)	8.8 (2.9)	7.9 (2.7)	0.475
**Post-experiment questionnaires**			
Fast Motion Sickness Scale (0–20)	0.2 (0.6)	3.2 (4.4)	**0.028**
Single-Item Measure of Presence (0–10)	7.3 (2.5)	6.6 (3.0)	0.560

Mean (± 1 SD) are indicated, with the exception of variables with a † symbol for which the number of participants is indicated, and § symbol for which the numbers represent the median. *P*-values for between-group comparisons are indicated when applicable and are in bold when < 0.05. VR – Virtual Reality; m/sTBI – Moderate-to-Severe Traumatic Brain Injury; PTA – Post-traumatic amnesia; GCS – Glasgow Coma Scale; ABC – Activities-Specific Balance Confidence Scale; CB&M – Community Balance and Mobility Scale; TUG – Timed Up and Go; MoCA – Montreal Cognitive Assessment; TMT – Trail Making Test

### Locomotor measures

Among the analyzed trials, six participants in each group contributed to a total of 16 collisions, accounting for 1.25% of the total trials. There were nine collisions observed in the m/sTBI group and seven in the healthy group. Seven of the total collisions occurred during ST walking, while 3 and 6 occurred during the simple and complex DT conditions, respectively. As indicated earlier, results below are for collision free trials.

**Fig. 2:**
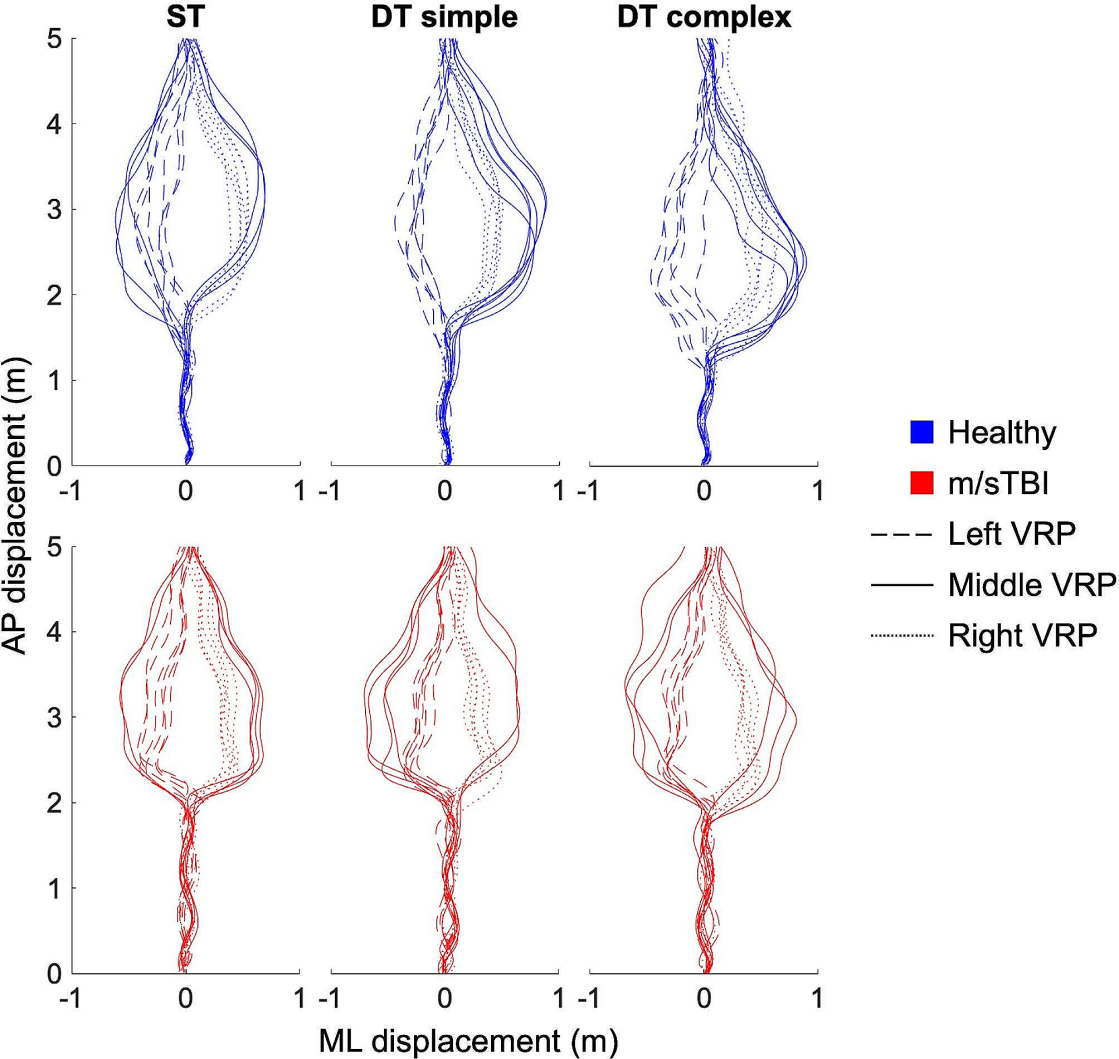
Walking trajectories of one healthy participant and one participant with m/sTBI for each locomotor task. Different scales were used for AP and ML displacement in order to better represent the walking trajectories. m/sTBI – Moderate-to-severe traumatic brain injury; AP – Antero-posterior; ML – Medio-lateral; VRP – Virtual pedestrian; ST – Single Task Walking; DT – Dual Task

In Fig. 2, representative walking trajectories from one healthy participant and one participant with m/sTBI are depicted for each locomotor condition. A noticeable modulation of the onset of trajectory deviation can be observed in the healthy participant across conditions, with a progressively earlier onset (or at a further distance from the VRP, not shown) during simple and complex DT conditions compared to ST walking. Such modulation, however, was less pronounced in the participant from the m/sTBI group. For the diagonal VRP approaches, it can also be observed that both representative participants consistently veered on the same side as the VRP was approaching from. In other words, they chose to pass behind the VRP, which was the case for 99% of trials when considering the whole sample of participants. When negotiating a VRP approaching from the middle, participants veered either right or left, with a preference to circumvent towards the right side (healthy group: 55.9%; m/sTBI group: 54.7%).

**Fig. 3:**
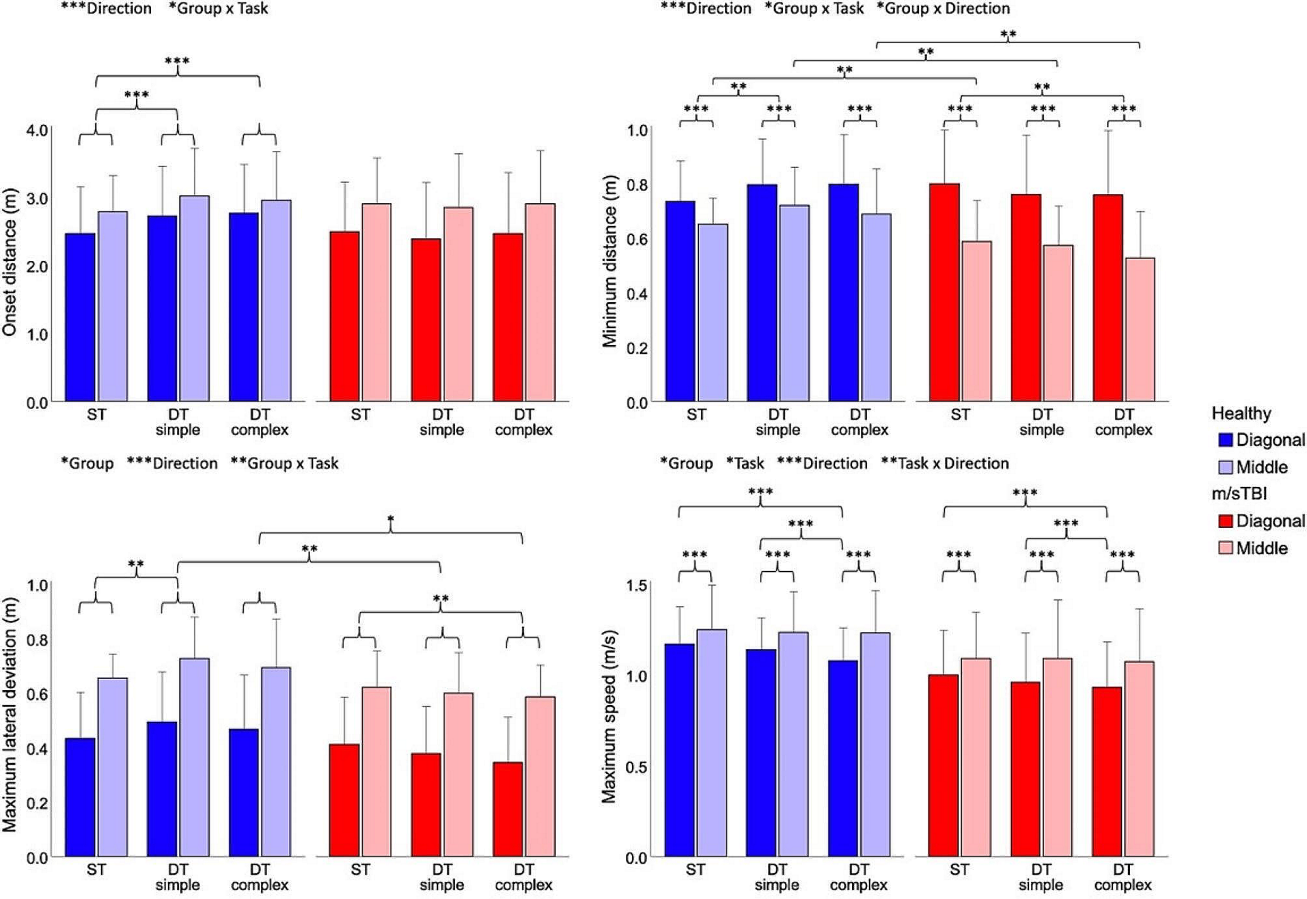
Locomotor measures (mean + 1 SD) for the healthy and m/sTBI participants across tasks and directions of pedestrian approach. Significant main and interaction effects are illustrated at the top of each graph, while results of post-hoc analyses are illustrated within each graph. m/sTBI – Moderate-to-severe traumatic brain injury; ST – single task walking; DT – dual task. Level of significance: *p-value < 0.05; **p-value < 0.01; ***p-value < 0.001

Results of the analyses of the anticipatory phase (onset distance of trajectory deviation), clearance phase (minimum distance and maximum lateral deviation) and maximum walking speed are illustrated in Fig. 3. In general, all measures were significantly modulated by the direction of approach of the VRP and most demonstrated an interaction effect between group and task. More specifically, onset distance of trajectory deviation exhibited a main effect of direction (X^2^(1, *N* = 1150) = 62.798, *p* < 0.001) and an interaction effect between group and task (X^2^(2, *N* = 1150) = 8.893, *p* = 0.012). Post-hoc analyses revealed that, overall, participants initiated their trajectory deviation at a greater distance from the VRP when circumventing a VRP approaching from the middle vs. diagonally (*p* < 0.001). Additionally, participants in the healthy group initiated their trajectory deviation at a greater distance from the VRP in both the simple (*p* < 0.001) and complex (*p* < 0.001) DT conditions vs. ST walking. Such effect of task complexity, however, was not present in the m/sTBI group (*p*-values: ST walking vs. DT simple = 0.362; ST walking vs. DT complex = 0.754). Of note, similar effects of direction (X^2^(1, *N* = 1150) = 4.646, *p* = 0.031) and group X task interaction (X^2^(2, *N* = 1150) = 17.424, *p* < 0.001) were observed for onset time of trajectory deviation (not shown in Fig. 3). In agreement with onset distance results, healthy participants were found to initiate their trajectory deviation earlier in both the simple (*p* < 0.001) and complex (*p* = 0.012) DT conditions vs. ST walking, while onset time values remained unchanged across levels of task complexity in the m/sTBI group (*p*-values: ST walking vs. DT simple = 0.025; ST walking vs. DT complex = 0.257).

For minimum distance, a main effect of direction (X^2^(1, *N* = 1271) = 27.015, *p* < 0.001) and interaction effects between group and task (X^2^(2, *N* = 1271) = 9.213, *p* = 0.010), as well as between group and direction (X^2^(1, *N* = 1271) = 4.244, *p* = 0.039), were found. Post-hoc analyses showed that individuals adopted smaller minimum distances from the VRP for the middle vs. diagonal VRP approaches (*p* < 0.001) but this difference was less pronounced in the healthy group than in the m/sTBI group (Healthy:$\varDelta$=0.09; m/sTBI:$\varDelta$=0.21 m, *p* < 0.001). Furthermore, while the healthy group increased their minimum distances in the simple DT walking condition compared to ST walking (*p* = 0.005), the m/sTBI group showed a reduction in minimum distance due to task complexity, with significantly smaller values for the complex DT condition vs. ST walking (*p* = 0.009).

The analysis of maximum lateral deviation revealed main effects of group (X^2^(1, *N* = 1150) = 5.322, *p* = 0.021) and direction (X^2^(1, *N* = 1150) = 259.389, *p* < 0.001), as well as an interaction effect between group and task (X^2^(2, *N* = 1150) = 10.779, *p* = 0.005). Post-hoc analyses demonstrated larger maximal lateral trajectory deviations for middle vs. diagonally approaching VRPs (*p* < 0.001). Between-group differences were further observed but only for DT walking conditions, with the healthy group displaying greater lateral deviation than the m/sTBI group (DT simple: *p* = 0.006; DT complex: *p* = 0.013). The influence of task complexity also differed between the two groups, as the healthy group displayed larger maximum lateral deviations in the simple DT walking vs. ST walking condition (*p* = 0.007) while the m/sTBI group showed smaller values in the complex DT walking vs. ST walking condition (*p* = 0.002).

Main effects of group (X^2^(1, *N* = 1266) = 4.355, *p* = 0.037), task (X^2^(2, *N* = 1266) = 6.607, *p* = 0.037) and direction (X^2^(1, *N* = 1266) = 43.480, *p* < 0.001), as well as an interaction effect between task and direction (X^2^(2, *N* = 1266) = 9.968, *p* = 0.007), were observed for maximum walking speed. Post-hoc comparisons showed that participants with m/sTBI adopted slower maximal walking speeds compared to the healthy group (*p* < 0.037), and smaller values were also observed for diagonally approaching VRP vs. those approaching from the middle (*p* < 0.001). Concerning the effect of task complexity, participants decreased their maximum speed during the complex DT condition vs. the two other conditions (simple DT: *p* < 0.001; ST walking: *p* < 0.001), but only for the diagonal approach. Although not illustrated in Fig. 3, minimum walking speed (X^2^(1, *N* = 1266) = 33.059, *p* < 0.001) and average walking speed (X^2^(1, *N* = 1266) = 79.148, *p* < 0.001) only showed a main effect of direction, whereby participants walked with slower minimum and average walking speeds when circumventing a VRP approaching from a diagonal direction vs. from the middle.

### Gaze behaviour measures

**Fig. 4:**
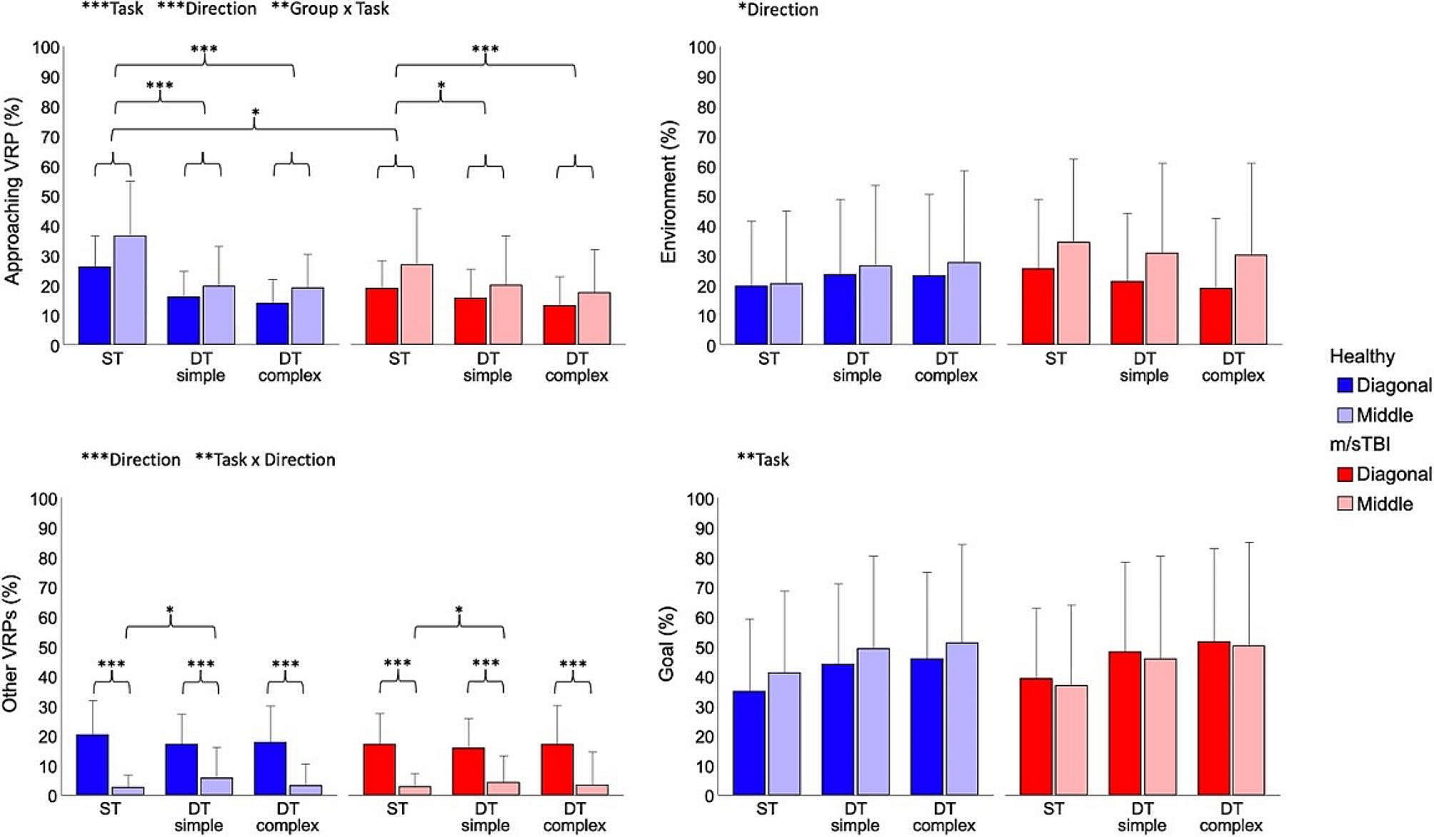
Gaze behaviour measures (mean + 1 SD) for the healthy and m/sTBI participants across tasks and directions of pedestrian approach. Significant main and interaction effects are illustrated at the top of each graph, while results of post-hoc analyses are illustrated within each graph. m/sTBI – Moderate-to-severe traumatic brain injury; ST – single task walking; DT – dual task. Level of significance: *p-value < 0.05; **p-value < 0.01; ***p-value < 0.001

Figure 4 illustrates the percentages of gaze fixation duration on the approaching VRP, the other VRPs, the goal and the rest of the environment. The percentage of gaze fixation duration on the approaching VRP was affected by the task (X^2^(2, *N* = 1217) = 75.463, *p* < 0.001) and direction of approach of the VRP (X^2^(1, *N* = 1217) = 18.868, *p* < 0.001), while showing an interaction effect of group and task (X^2^(2, *N* = 1217) = 11.301, *p* = 0.004). Post-hoc analyses showed that participants gazed on the approaching VRP for longer durations during the ST walking condition vs. both the simple (*p* < 0.001 for the healthy group and *p* = 0.023 for the m/sTBI group) and complex (*p* < 0.001) DT conditions, as well as when the approaching VRP was coming from a middle direction vs. diagonal (*p* < 0.001). The between group differences were more pronounced in the ST walking (*p* = 0.022) where the healthy group gazed upon the approaching VRP 8.5% more than the m/sTBI group. As for the percentage of gaze fixation directed toward the other VRPs, it showed a main effect of direction (X^2^(1, *N* = 1217) = 208.985, *p* < 0.001) and an interaction effect of task and direction (X^2^(2, *N* = 1217) = 9.010, *p* = 0.011). Overall, participants gazed at other VRPs for longer durations when the approaching VRP was coming from a diagonal vs. the middle direction (*p* < 0.001). The task and direction interaction effect were explained by a small but significant increase in the percentage of gaze fixation on other VRPs during the simple DT condition vs. ST walking (*p* = 0.017) for the middle approach only.

The percentage of gaze fixation on the environment was only affected by the direction of VRP approach (X^2^(1, *N* = 1217) = 10.662, *p* = 0.001), with participants gazing at the environment for longer durations while avoiding a middle vs. a diagonally approaching VRP. As for the percentage of gaze fixation on the goal, it was only affected by the task (X^2^(2, *N* = 1217) = 13.102, *p* = 0.001), with smaller values for ST walking vs. both the simple (*p* = 0.002) and complex DT (*p* < 0.001) conditions.

### Cognitive measures

All participants made errors in at least one of the cognitive tasks. The perceived level of difficulty on the cognitive tasks was significantly higher in the m/sTBI group than in the healthy control group for all conditions (cognitive ST simple: m/sTBI = 3.76 ± 2.59, healthy = 0.92 ± 1.14, *p* = 0.002; cognitive ST complex: m/sTBI = 4.86 ± 2.75, healthy = 2.06 ± 1.83, *p* = 0.008; DT simple: m/sTBI = 4.03 ± 2.68, healthy = 1.48 ± 1.28, *p* = 0.007; DT complex: m/sTBI = 5.47 ± 2.12, healthy = 2.00 ± 1.42, *p* = 0.000). We observed twice as many participants making errors in the m/sTBI group vs. the healthy group for the simple cognitive ST condition (*n* = 8 vs. 4), the complex cognitive ST condition (*n* = 12 vs. 6) and the simple DT condition (*n* = 12 vs. 6). For the complex DT condition, the number of individuals making errors was the same between groups (11 for each).

**Fig. 5:**
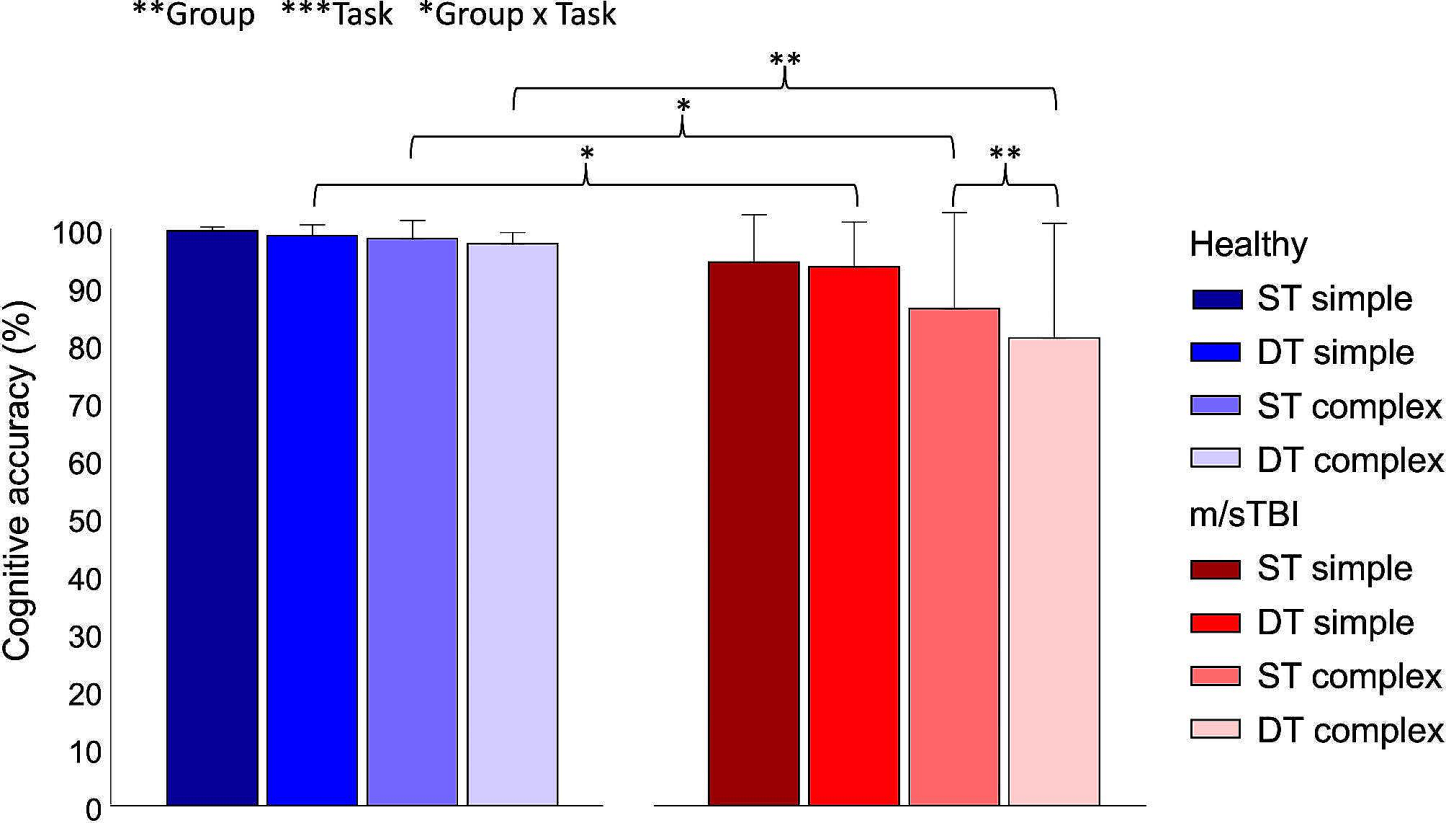
Percentages of correct response (mean + 1 SD) on the simple and complex cognitive tasks performed in single- and dual-task conditions in healthy and m/sTBI participants. m/sTBI – Moderate-to-severe traumatic brain injury; ST – cognitive single task; DT – dual task. Level of significance: *p-value < 0.05; **p-value < 0.01; ***p-value < 0.001

As a whole, participants demonstrated proportions of correct response on the cognitive tasks that ranged from 81.0% (DT complex for the m/sTBI group) to 99.6% (cognitive ST simple for the healthy group), as depicted in Fig. 5. Statistical analyses revealed a main effect of group (X^2^(1, *N* = 96) = 8.060, *p* = 0.005) and task (X^2^(3, *N* = 96) = 16.950, *p* < 0.001), as well as an interaction effect between group and task (X^2^(3, *N* = 96) = 8.029, *p* = 0.045). Except for the simple cognitive ST condition, the healthy group showed higher proportions of correct response than the m/sTBI group on all task conditions (DT simple, *p* = 0.014; cognitive ST complex, *p* = 0.01; DT complex, *p* = 0.003). In addition, only the m/sTBI group experienced a decrease in performance in the complex cognitive task performed in single vs. dual-task condition (*p* = 0.008).

### Dual-task costs

The analyses of dual-task costs (DTCs) related to locomotor, gaze behaviour, and cognitive measures are summarized in Table 2. In general, statistically significant main effects of group and/or complexity were identified, but no statistically significant interactions between group and complexity were found for any of the DTC measures (*p*-value = 0.109–0.935). Regarding locomotor DTCs, significant main effects of group emerged for onset time (X^2^(1, *N* = 48) = 10.260, *p* = 0.001), onset distance (X^2^(1, *N* = 48) = 10.347, *p* = 0.001), minimum distance (X^2^(1, *N* = 48) = 10.140, *p* = 0.001), and maximum lateral deviation (X^2^(1, *N* = 48) = 10.381, *p* = 0.001). In fact, while the healthy group displayed negative DTCs for all of those measures, the m/sTBI group instead showed relatively small but positive DTCs. In practical terms, this means that healthy participants performed a trajectory deviation earlier and at a greater distance from the VRP, while increasing their minimum distance and maximum lateral deviation during dual- vs. single-task conditions. Conversely, the m/sTBI group initiated a trajectory deviation later and a closer distance from the VRP and showed reduced minimum distance and maximum lateral deviation in the dual- vs. the single-task condition, although those changes were generally of small magnitude. A main effect of task complexity was also identified for both maximum speed (X^2^(1, *N* = 48) = 6.626, *p* = 0.01) and maximum lateral deviation (X^2^(1, *N* = 48) = 7.434, *p* = 0.006). For both of these measures, an increase towards a more positive cost was observed in the complex vs. simple DT condition, implying a reduction in maximum speed and maximum lateral deviation in the complex vs. simple DT condition.

**Table 2:** Dual-task costs

Locomotor DTCs (%)	Group				*P*-value	
	Healthy		m/sTBI			
	Complexity					
	Simple	Complex	Simple	Complex	Group	Complexity
Onset time	-9.81 (10.22)	-7.79 (9.51)	5.49 (11.70)	4.53 (19.03)	**0.001**	0.850
Onset distance	-10.11 (11.21)	-10.03 (9.10)	1.50 (13.89)	2.12 (15.32)	**0.001**	0.915
Minimum distance	-9.24 (12.12)	-7.71 (14.70)	4.22 (12.01)	6.43 (9.26)	**0.001**	0.343
Maximum speed	1.84 (7.00)	5.42 (10.14)	3.08 (11.37)	5.19 (15.21)	0.906	**0.010**
Maximum lateral deviation	-12.57 (16.20)	-5.32 (15.22)	3.28 (15.66)	11.48 (11.97)	**0.001**	**0.006**
**Gaze behaviour DTCs (%)**						
Approaching VRP	42.29 (9.87)	46.95 (10.66)	20.62 (32.53)	33.11 (28.63)	**0.033**	**0.011**
Other VRPs	4.08 (20.98)	6.98 (25.53)	-5.90 (50.07)	-14.63 (56.20)	0.302	0.578
Goal	-32.72 (62.17)	-33.35 (39.16)	-27.52 (60.83)	-42.79 (79.99)	0.923	0.486
**Cognitive DTCs (%)**						
Cognitive accuracy	0.79 (1.48)	0.78 (4.56)	0.62 (6.01)	6.91 (9.82)	**0.038**	0.110

Mean (1SD) for dual-task costs. DTC – Dual-task cost; VRP – Virtual pedestrian. Statistically significant *p*-values for the effect of group and complexity are shown in bold

Gaze behaviour measures showed the largest DTCs amongst all measures, reaching values as high as 47% and − 43%, respectively, for gaze fixation duration on the approaching VRP and the goal. While both groups exhibited a positive DTC for gaze fixation duration on the approaching VRP, this DTC was significantly smaller (X^2^(1, *N* = 46) = 4.572, *p* = 0.033) in the m/sTBI group than in the healthy group. In other words, while dual tasking induced shorter fixation durations on the approaching VRP, such reduction was significantly less pronounced in the m/sTBI group than in the healthy group. Of note, negative DTCs were observed for gaze fixation on the goal and, to some extent on the other VRPs (as seen in the m/sTBI group), indicating that participants fixated more on those elements in DT conditions. For the latter two measures, however, there were no differences between groups. A significant main effect of complexity was observed in relation to DTC in gaze fixation duration on the approaching VRP (X^2^(1, *N* = 46) = 6.405, *p* = 0.011). The cost was higher in the complex vs. simple dual-task condition ($\varDelta$=8.57%), which means that the reduction in gaze fixation duration on the approaching VRP was more pronounced for the complex vs. simple dual-task condition.

Lastly, the m/sTBI group exhibited positive DTCs in cognitive task accuracy reaching at most 6.91%, indicating a modest overall reduction in cognitive performance in dual-task conditions, while values for the healthy controls remained close to zero (0.78–0.79%). A statistically significant main effect of group was observed for DTC in cognitive accuracy (X^2^(1, *N* = 48) = 4.293, *p* = 0.038), with the m/sTBI group experiencing a larger DTC compared to the healthy group. Task complexity did not significantly impact this measure.

## Discussion

This study was the first to examine the dual-task locomotor and cognitive costs in individuals with chronic m/sTBI simultaneously performing a pedestrian collision avoidance task and an auditory cognitive task within a virtual community environment. The individuals with m/sTBI recruited in this study showed, as expected [36], alterations in their clinical tests of balance and mobility and executive functions. However, they were still relatively high functioning and walked on average at a comfortable speed of 1.2 m/s, which is equal or greater than the speed required for independent community ambulation [50, 70, 71]. Yet, when exposed to a complex walking task such as avoiding a collision with pedestrians approaching from different directions, they did show differences in their collision avoidance strategies compared to healthy controls. Furthermore, under dual-task conditions, they not only showed a mutual cognitive-locomotor interference that contrasted with the single locomotor interference observed in the healthy individuals, but they also adopted collision avoidance strategies that markedly differed from that of their healthy counterpart.

### Dual task-induced adaptations in collision avoidance strategy

The present study highlights between-group differences in both the anticipatory and clearance phases when executing the collision avoidance task under dual-task conditions. Healthy controls exhibited slower walking speeds, earlier and more distant onsets of trajectory deviation in the anticipatory phase, along with increased maximum lateral deviations and larger minimum distances in the clearance phase for the dual- vs. single-task conditions. Individuals with m/sTBI also walked slower for the dual-task conditions, but they maintained similar distances and times at onset of trajectory deviation between dual- and single-task conditions, indicating a lack of modulation of the anticipatory phase of obstacle circumvention. This lack of modulation likely resulted in the closer proximity to pedestrians observed in the clearance phase for this group when dual tasking, which was reflected by smaller maximum lateral deviations and minimum distances.

The dual-task adaptations observed in healthy controls apparently contrast with a recent study by Bhojwani et al. (2022), which used a protocol similar to the one used in the present study [45] and where no dual-task adaptations in terms of locomotor measures were observed. The healthy individuals included in the present study, however, were aged-matched to the m/sTBI group and older (average 41.8 years, range 24–53) than those tested earlier (24.9 years, range 18–29) [45]. Age-related changes in sensorimotor and cognitive functions [72–74], as well as in dual-task walking abilities [75, 76], likely explain differences between the two studies. Earlier onsets of trajectory deviations [18, 68], but also larger minimum distances [23] were observed in previous obstacle circumvention studies when participants were exposed to riskier or unfamiliar obstacle conditions, suggesting the use of a conservative circumvention strategy. Such adaptations on the part of healthy individuals in the present study may thus reflect the use of a conservative or safer avoidance strategy, which aimed to minimize the risk of collision as cognitive resources were drawn upon to complete the concurrent cognitive task.

A similar dual-task induced conservative behaviour was recently reported in the context of an obstacle circumvention study involving individuals with Parkinson’s disease and age-matched healthy individuals [77]. However, individuals from the m/sTBI group in the present study seemed instead to exert a riskier collision avoidance behaviour, as they failed to modulate the onset of trajectory deviation and adopted smaller obstacle clearances under dual-task conditions. Past studies related to obstacle crossing (stepping over) under dual-task conditions have reported both reduced [42] and increased toe clearance [37] in individuals with m/sTBI. While reasons for such differences between studies remain unclear, the task involved in the present study, where participants circumvented moving interferers approaching from different directions, is more demanding than stepping over a static obstacle in terms of planning and execution. It would thus require more cognitive resources, possibly exceeding a total cognitive capacity that is already compromised after m/sTBI and resulting in a riskier as opposed to a safer collision avoidance behaviour.

Given the divergent dual-task induced locomotor adaptations between the two groups, it is not surprising that the groups exhibited DTCs of different polarity (i.e., negative vs. positive DTCs) for most locomotor measures. Those positive and negative DTCs reflect, respectively, a decrease and an increase in a given measure under dual-task conditions. It is important to note, however, that some DTC values in the m/sTBI group were modest (e.g., 1.5% and 2.12% for onset distance), reflecting a lack of adaptation rather than an actual change. Overall, such alterations in the pattern of DTCs in the individuals with m/sTBI did not result in more collisions. A heightened risk of collisions in busier community environments (e.g., shopping mall with multiple pedestrians walking in different directions), however, cannot be excluded.

Interestingly, the measures that displayed the largest DTCs in the present study were those related to gaze behaviour. Indeed, both groups showed a marked reduction in gaze fixation on the approaching VRP and an increased gaze fixation on the goal during dual-task conditions. Several studies have suggested gaze behaviour to be an indicator of attention allocation, with longer and/or more frequent fixations being devoted to objects or cues that are being focussed on [8]. In the present study, the fact that a non-visually based cognitive task modified the relative gaze fixation duration on visual cues (i.e., approaching VRP and goal) that are essential for the successful completion of the locomotor task suggests an interference with the allocation of attention. Yet, as participants did not experience more collisions under dual-task conditions, it is possible that quickly looking at the approaching pedestrian was sufficient to make the proper adjustments in the locomotor trajectory and avoid a collision. Other elements such as peripheral vision [78, 79] and eye proprioceptive information provided through gaze shifts [80] may have further assisted with the localization of the interferer. As for the prolonged fixation on the goal, it may have served the purpose of fulfilling the goal-oriented component of the walking task [45]. It is also possible that in response to the increased attentional load, individuals reduced their visual scanning of the environment, resulting in a gaze orientation towards the midline where the goal was located. This hypothesis, however, would need to be verified through spatial-temporal analysis of gaze allocation on the different features present in the virtual simulation.

The reduction in gaze fixation on the approaching pedestrian in dual-task conditions decreased the DTC by up to a factor of two for the m/sTBI group compared to the healthy group. As suggested earlier, it might be expected that individuals with m/sTBI would be more reliant on visual information than healthy individuals to perform an obstacle avoidance task while walking [42]. In the present study and for the single task condition, however, it is likely that they focussed on other elements in the environment than the approaching VRP, although none of those other elements (goal, environment, other pedestrians) considered in isolation came out as significantly different between the two groups. Whether they were focusing on other visual cues that helped them planned their trajectory or on other elements of the scene that were irrelevant to the task (e.g., buildings, trash bins, etc.) remains an open question. As for the effects due to dual-tasking compared to single tasking, it is possible that the m/sTBI group, who already showed short visual fixations on the approaching VRP in single-task walking, could not afford to reduce this gaze fixation to the same extent as healthy controls in the dual-task conditions, in order to provide adequate visual information about the obstacle and successfully complete the collision avoidance task.

The complexity of the cognitive task had overall a minimal impact on locomotor DTCs, except for maximum walking speed and maximum lateral deviation, for which both groups increased their DTCs towards more positive values in the more complex dual-task condition. This indicates that individuals from both groups experienced slower maximum walking speeds and less maximum lateral deviations in the complex vs. simple dual-task condition. Such findings are consistent with the larger DTCs observed for various locomotor measures due to increased task complexity in other populations such as stroke [21, 81]. Such effect of task complexity is likely due to participants’ cognitive resources being further ‘taxed’ when performing the more complex cognitive task.

Present findings also showed that the direction of obstacle approach modulated locomotor measures. Such modulation, observed in earlier studies, is shown to be characterized by one or several of the following changes, including earlier onsets of trajectory deviation, smaller minimum distances, greater maximum lateral displacements, faster walking speeds and longer durations of gaze fixation on the approaching VRP in the presence of obstacles/interferers approaching from the middle vs. diagonally [6, 30, 45, 68]. It was suggested that negotiating with a middle obstacle approach is more challenging than with a diagonal approach, as the former absolutely requires a trajectory change to prevent a collision, whereas the latter can also be avoided through walking speed adjustments [6, 25, 45]. In the present study, an interaction of group and direction also indicated that individuals with m/sTBI maintained smaller minimum distances from the interferer compared to healthy controls for the middle approach specifically, both in single and dual-task conditions. This illustrates the increased difficulty experienced by individuals with m/sTBI in negotiating this more challenging or riskier obstacle condition.

### Dual task cognitive performance

Individuals with m/sTBI showed alterations in both locomotor and cognitive performances during dual-task walking, in contrast to healthy controls whose alterations in performance were limited to the locomotor task. Although not statistically different from the simple dual-task condition, the cognitive DTC in the m/sTBI group was especially pronounced for the complex dual-task condition. These findings, which suggest the presence of a cognitive-motor interference, align with previous studies carried out in individuals with m/sTBI [37, 42] and other populations with neurological conditions such as stroke [21, 28, 32]. They also align with the fact that individuals with m/sTBI in the present study gave higher subjective ratings of task difficulty for the dual-task condition than for the single-task condition, and generally higher ratings than healthy controls.

Altered executive functions could explain, at least in part, this DTC in cognitive performance, as individuals with m/sTBI exhibited a reduced performance on clinical tests of cognitive executive functions to start with, as well as on the Auditory Stroop Task performed as a single task. In fact, individuals with m/sTBI can experience a variety of cognitive deficits, including slowed information processing, impaired long-term memory, attention, working memory, executive function, mental flexibility, inhibitory control and mental fatigue [82]. Thus, while individuals with m/sTBI may possess sufficient cognitive abilities to successfully perform simple cognitive tasks under single-task conditions, their performance is compromised when exposed to cognitive tasks with higher demands in terms of executive functions, or when attention is divided as in dual-task walking. More specifically, alterations in visuospatial processing, as indicated in the present study by results of m/sTBI participants on TMT-A and TMT-B, could be at cause, since such alterations were shown to correlate with smaller toe clearance when stepping over an obstacle under both single and dual-task conditions in individuals with m/sTBI [83]. 

As for the healthy individuals in the present study, who were in their middle adulthood, they presented a locomotor-only interference that contrasts with the cognitive-only interference previously reported for healthy young adults tested under identical experimental conditions [45]. In fact, their levels of accuracy on the cognitive tasks (97.30 to 99.55%) appear to surpass that of the healthy young adults (range 88.53 to 97.65) [45], with a 9% difference in accuracy on the complex dual-task condition. This enhanced performance on the cognitive task, along with the locomotor-only interference, suggest that the middle-aged adults in the present study were more focused on the cognitive tasks at the expense of the locomotor task, for which they showed a more conservative behaviour.

### Clinical implications

Our study indicates that individuals with a chronic m/sTBI, in spite of a good locomotor recovery, exhibit residual deficits in obstacle circumvention that are especially pronounced in dual-task conditions, possibly increasing the risk of collisions and interfering with community walking abilities. Dual-task walking training could be integrated early in the process of rehabilitation, while exposing individuals to scenarios of various complexities that better simulate real-life locomotor challenges. Additionally, and as observed in a recent study involving stroke survivors [81], our study revealed that standardized clinical assessments such as TUG-Cog may overlook dual-task walking difficulties related to complex daily locomotor tasks. Such observation highlights the potential of VR as a tool for the evaluation and training of dual-task walking abilities in individuals with m/sTBI.

### Limitations

We acknowledge some limitations in our study. This protocol was conducted during the Covid-19 pandemic, and therefore participants circumvention strategies may have been influenced by the unique context of social distancing and heightened anxiety levels [68]. Such an effect, however, would be expected to be similar in both groups. In addition, the use of a VR-based protocol may have impacted on the locomotor behaviour, inducing larger obstacle clearances and slower walking speeds compared to what would be observed in the real world; these differences, however, were shown to be of small magnitude (10–13%) [6]. Furthermore, the ability to control experimental conditions and the safety of VR outweigh this limitation, making it a valuable tool for studying complex locomotor tasks.

## Conclusion

This study revealed that individuals with chronic m/sTBI present alterations in both locomotor and cognitive performances when circumventing pedestrians under dual-task conditions, as opposed to healthy individuals who only show an alteration in their locomotor performance. In addition, the nature of the dual-task induced alterations differed between groups, the m/sTBI group showing a riskier collision avoidance behaviour that contrasts with the more conservative locomotor behaviour displayed by healthy individuals. The extent of gaze behaviour modulation under dual-task condition also differed between the two groups, possibly reflecting alterations in the allocation of attention. Present findings raise concerns about potential collisions in crowded community environments in individuals with m/sTBI, while highlighting the compromised complex walking abilities in this population who otherwise present a good locomotor recovery. Collectively, these findings emphasize the need to assess and enhance these abilities as integral components of rehabilitation.

## Data Availability

The datasets used and/or analysed during the current study are available from the corresponding author on reasonable request.
